# Molecular characterization of group A rotaviruses in Mukuru slums Kenya: detection of novel strains circulating in children below 5 years of age

**DOI:** 10.1186/s13104-017-2611-z

**Published:** 2017-07-17

**Authors:** Joshua Ndung’u Gikonyo, James Nyangao, Cecilia Mbae, Carlene Sang, Eliud Njagi, Joseph Ngeranwa, Mathew Esona, Mapaseka L. Seheri, Grace W. Gitau, Kedra Raini, Samuel Kariuki

**Affiliations:** 1 Department of Biochemistry and Biotechnology, School of Pure and Applied Sciences, Kenyatta University, P.O. Box 43844-00100, Nairobi, Kenya; 2 Kenya Medical Research Institute, P.O. Box 43640-00100, Nairobi, Kenya; 3 Department of Biochemistry and Biotechnology, Technical University of Kenya, P.O. Box 52428-00200, Nairobi, Kenya; 4 Centre for Disease Control and Prevention, Greater Atlanta Area, Atlanta, USA; 5 Diarrheal Pathogens Research Unit, University of Limpopo, Medunsa Campus, Polokwane, South Africa

**Keywords:** Rotavirus, Genotyping, Viral protein 4, Viral protein 7

## Abstract

**Background:**

Gastroenteritis is a public health concern due to high morbidity and mortality among children. Rotaviruses are the leading etiological agents of severe gastroenteritis in children and accounts for more than half a million deaths per year in Africa. The study aimed at investigating the rotavirus genotypes that were circulating in children aged 5 years and below in and around Mukuru slums in Nairobi County Kenya.

**Methods:**

A purposive cross sectional sampling method was applied where 166 samples were collected from children below 5 years of age and taken to Kenya Medical Research Institute virology laboratory. Presence of rotaviruses was determined using reverse transcription polymerase chain reaction, while extraction was done using ZR Soil/Fecal RNA MicroPrep™ extraction kit. This was followed by reverse transcription and genotyping using various group A rotavirus primers.

**Results:**

The G type was successfully determined in 37 (92.5%), while the P type was successfully determined in 35 (87.5%) of the 40 (24%) page positive samples. Type G1 was the most predominant of the G types (40.5%), and the incidences of G3 and G9 were 21.6 and 32.4% respectively. Mixed types G3/G9 were detected at 5.4%. Three P types existed in Mukuru slums, P[8] (60%), P[6] (22.9%), P[4] (11.4) and their relative incidence varied over the 15 months of this study.

**Conclusions:**

The G types and P types detected in this study are important causes of acute gastroenteritis in Mukuru slums Nairobi Kenya. An indication that the prevalence of certain genotypes may change over a rotavirus season is significant and mirrors observations from studies in other tropical climates. Thus monitoring of the genotypic changes among circulating viruses should be encouraged over the coming years.

## Background

Rotavirus is the leading cause of severe dehydrating diarrhoea in children under 5 years of age worldwide, and accounts for around half a million deaths in infants in developing countries per year [1]. Even in advanced countries such as the US, it accounts for up to 500 deaths per annum [2]. Rotaviruses are the leading cause of stomach flu among children between 3 and 15 months of age and are associated with more than half a million deaths per year in Africa and Asia [3]. Worldwide, rotaviruses cause approximately 112 millions of domestic episodes of diarrhoea, 25 millions of clinic visits, 2 millions of hospitalizations and about 800,000 deaths of children below 5 years of age annually [4].

Rotavirus is a segmented double stranded RNA (dsRNA) virus, and presents a triple concentric layer of proteins. Its genome is made up of 11 segments of double stranded RNA held in the inner core of the three-layered virus [5]. The genome codes for six viral structural proteins (VP1, 2, 3, 4, 6, 7) and six non-structural proteins (NSP1-6).

Because the genes encoding these proteins segregate independently of each other during reassortment, a dual-serotyping system to account for the specificities of both VP7 and VP4 has been adopted. Thus the classification of rotaviruses is based on differences in the VP7 (G) and VP4 (P) capsid proteins, where the G serotypes 1–4, and P genotypes P[8] and P[4] predominate worldwide [6]. Globally, viruses carrying the genotype pairs G1 P[8], G2 P[4], G3 P[8], and G4 P[8] are important causes of diarrhoea among infants worldwide, accounting for 95.9% of all typeable strains [7].

Polyacrylamide gel electrophoresis (PAGE) of the rotavirus RNA genome allows detection and classification of the viruses into two major groups; the long (L) and the short (S) electrophoretic profiles based on the migration patterns of gene segments 10 and 11 on polyacrylamide gel. The variations in the electrophoretic mobility of one or more RNA segments allow different rotavirus strains to be further classified into seven electropherotypes (e-type) from A–G, according to the migration pattern of the 11 RNA segments [8]. Electrophoresis of the rotavirus RNA genome has often been used as a useful indicator of the genomic diversity of rotavirus isolates in populations over a certain period [9].

Given the paucity of data regarding the molecular epidemiology of rotaviruses in the developing countries, the aim of this study was to determine the genotypes of group A rotaviruses circulating in children aged 5 years and below, within and around Mukuru slums in Nairobi County.

## Methods

From January 2010 to March 2011, 166 fecal samples were collected from outpatient children aged 5 years and below, seeking treatment for diarrhoea at St. Mary’s health centre and Reuben medical centre, both located at different regions in Mukuru slums.

The samples were collected by the technicians at the health centre’s laboratory. The demographic characteristics of the patient and a clinical history for each patient were collected by a nurse or attending physician, and the following signs and symptoms were noted: diarrhoea, vomiting, abdominal pain, dehydration and fever. Specimens were transported to the Centre for Viral Research Laboratory in Kenya Medical Research Institute in sterile cryovials placed in a cooler and stored at −20 °C.

The presence of rotavirus in stool samples was determined using ELISA techniques. Genotyping involved polyacrylamide gel electrophoresis of rotavirus genomic RNA, which determined the percentage of the long and the short rotavirus electropherotypes present. This was followed by reverse transcription of purified rotavirus dsRNA (VP4 and VP7 cDNA synthesis), amplification of cDNA by multiplex PCR, and a nested PCR of the amplified VP4 and VP7 genes.

The VP7 strains were genotyped using a reverse transcriptase (RT)-PCR method described by Gouvea et al. [10], using a cocktail of VP7 specific primers to the six human serotypes (G_1_, G_4_, G_8_ and G_9_). VP4 specificity was identified using the RT-PCR method described by Gentsch et al. [7], similarly using a nested PCR reaction with a mixture of VP4 specific primers.

### Data analysis

The data collected was routinely entered into a database created in Microsoft excel for analysis, while statistical analysis was done using the chi squire. The prevalence and seasonal distribution of different enteric viruses, age and gender of the child were presented as proportions of the total population.

## Results

### Electropherotypes

**Fig. 1:**
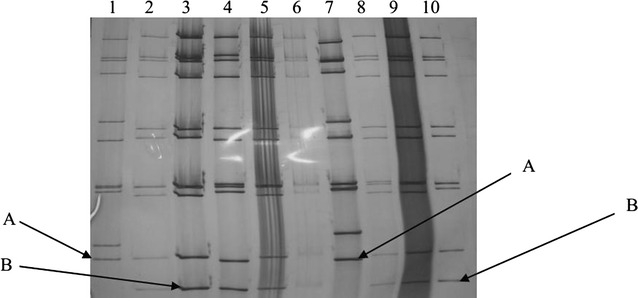
Genomic RNA electrophoresis of representative strains from rotavirus electropherotypes identified in Mukuru slums. *Letter A* indicates a short electropherotype while *B* indicates a long electropherotype. RNA migration was from the *top* to the *bottom*, and *lanes 2*, *3*, *4*, *5*, *6*, *8*, *9*, and *10* represents long electropherotypes, while *lanes 1* and *7* represent the short electropherotypes detected

A total of 166 fecal samples were collected and tested for rotavirus. Out of these, 40 (24%) were rotavirus-positive (by ELISA). They were ran through PAGE and yielded rotavirus RNA electrophoretic patterns. Thirty-two (80%) of the isolated strains were long electropherotypes while eight (20%) of the strains were short electropherotypes. There were no profiles of mixed infections detected in the electropherotypes (Fig. 1).

**Fig. 2:**
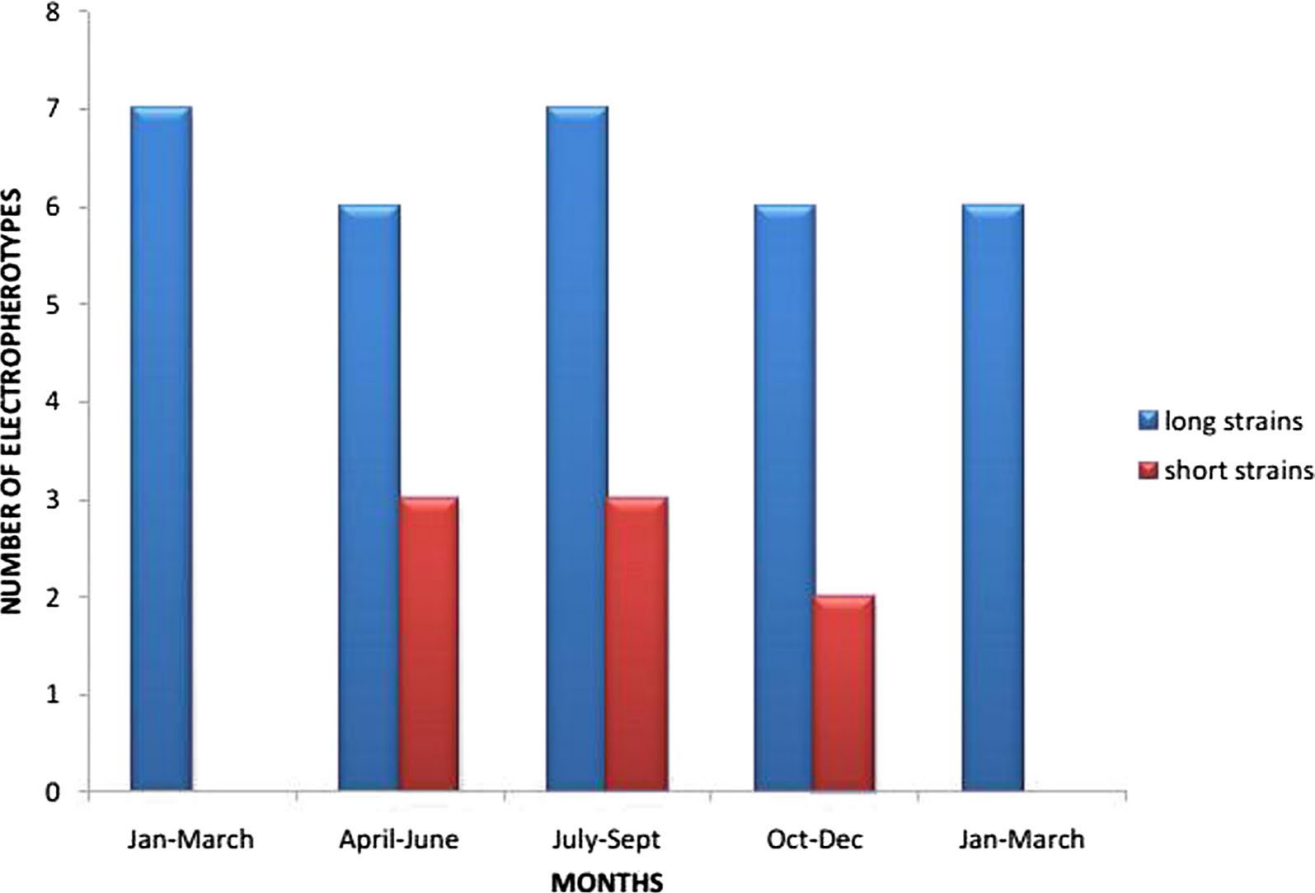
Temporal distribution of rotavirus electropherotypes in January 2010 through March 2011 in Mukuru slums Nairobi. The *red columns* represent the short electropherotypes while the *blue columns* represent the long electropherotypes

All along the 15 months of this study, two different rotavirus strains as defined by PAGE-RNA electropherotypes co-circulated in the Mukuru slums, with the long strain being predominant and persistent during the period of this survey (Fig. 2). Throughout the study, the long electropherotypes were always associated with G1 and G9 genotypes, whereas the short electropherotypes were associated with genotype G3. However, it was noted that the pattern of the electropherotype suggested but did not confirm a particular genotype.

The monthly distribution of the long electropherotypes appeared to be random and no apparent seasonal variations could be detected. The short profiles were more prevalent in cold and wet months and were not observed in dry months of the study period (Fig. 2).

The distribution of long RNA electropherotypes did not appear to vary with age and it occurred in all ages under study (1 month to 5 years old), while the short RNA profiles were identified among children aged up to 2 years old (Table 1).

**Table 1:** Distribution of rotavirus RNA electropherotypes among different age groups in Mukuru slums Nairobi

Age groups (months)	Electropherotypes	
	Long patterns	Short patterns
0–3	7	2
4–6	5	2
7–12	7	3
13–24	6	1
25–60	7	0

### VP7 genotypes

**Fig. 3:**
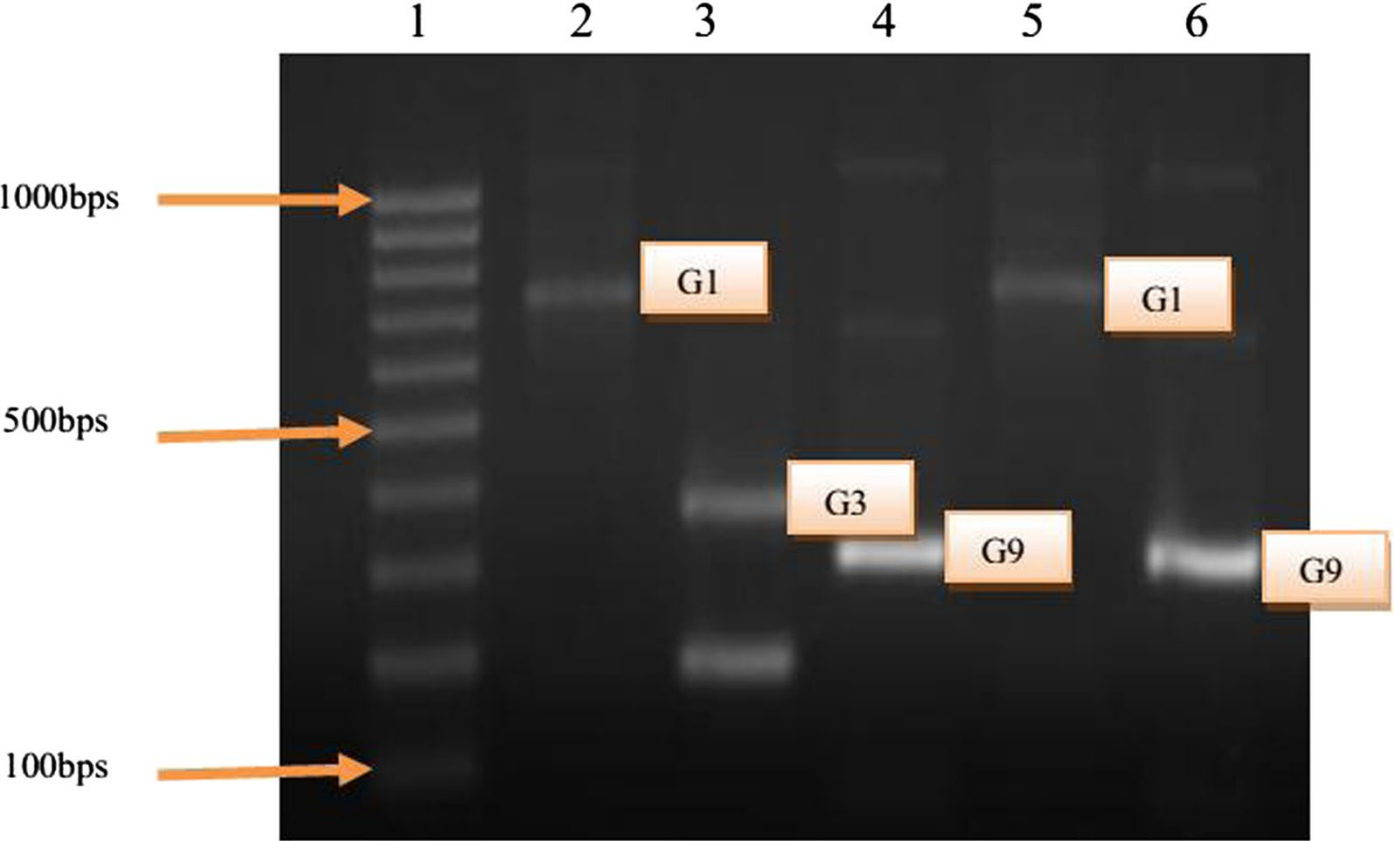
VP7 genotypes as seen under UV light in agarose gel. *Lane 1* 1000 bp marker, *lane 2* G1 (749 bp), *lane 3* G3 (374 bp), *lane 4* G9 (306 bp), *lane 5* G1 (749 bp) and *lane 6* G9 (306 bps)

The G types (VP7 associated) were successfully determined in 37 (92.5%) of the 40 page positive samples. A G type was assigned after a PCR with G1, G2, G3, G4, G8 and G9 specific primers was carried out on rotavirus cDNA (Fig. 3). The overall incidence for G typing was G1, 40.5% (15), followed by G3, 21.6% (8), and G9, 32.4% (12) (Table 2). Mixed types (G3/G9) were detected at 5.4% (2), while G2, G4 and G8-type viruses were not detected. The remaining three (7.5%) positive samples although positive for rotavirus, remained untypable (Table 2). The incidence of each type was seen to vary from month to month. G1 and G9 occurred most frequently in February and August, while G4 was high in November and December.

**Table 2:** Distribution of rotavirus G and P types over 15 months in Mukuru slums

	No. and % (in parenthesis) of G types						No. and % (in parenthesis) of P types					
	G1	G3	G9	Mix^α^	NT	Total	P4	P6	P8	Mix^α^	NT	Total
Jan–March 2010	3 (30)	2 (20)	4 (40)	1 (10)	0	10	0	3 (33.3)	5 (55.6)	1 (11.1)	0	9
April–June 2010	2 (33.33)	1 (16.7)	2 (33.33)	0	1 (16.7)	6	1 (16.7)	1 (16.7)	3 (50)	0	1 (16.7)	6
July–Sept 2010	6 (54.6)	2 (18.2)	1 (9.1)	1 (9.1)	1 (9.1)	11	1 (10)	1 (10)	6 (60)	1 (10)	1 (10)	10
Oct–Dec 2010	2 (40)	1 (20)	1 (20)	0	1 (20)	5	0	1 (25)	3 (75)	0	0	4
Jan–March 2011	2 (25)	2 (25)	4 (50)	0	0	8	2 (25)	2 (25)	4 (50)	0	0	8
Total	15	8	12	2	3	40	4	8	21	2	2	37

*Mix* ^α^ mixed G or P types, *NT* not typeable, *in brackets* %

### VP4 genotypes

The P types (VP4 associated) were successfully determined in 35 (87.5%) of the 40 page positive samples (Table 2). A P type was assigned after a PCR with P[4], P[6], P[8], P[9] and P[10] specific primers was carried out on rotavirus cDNA template.

**Fig. 4:**
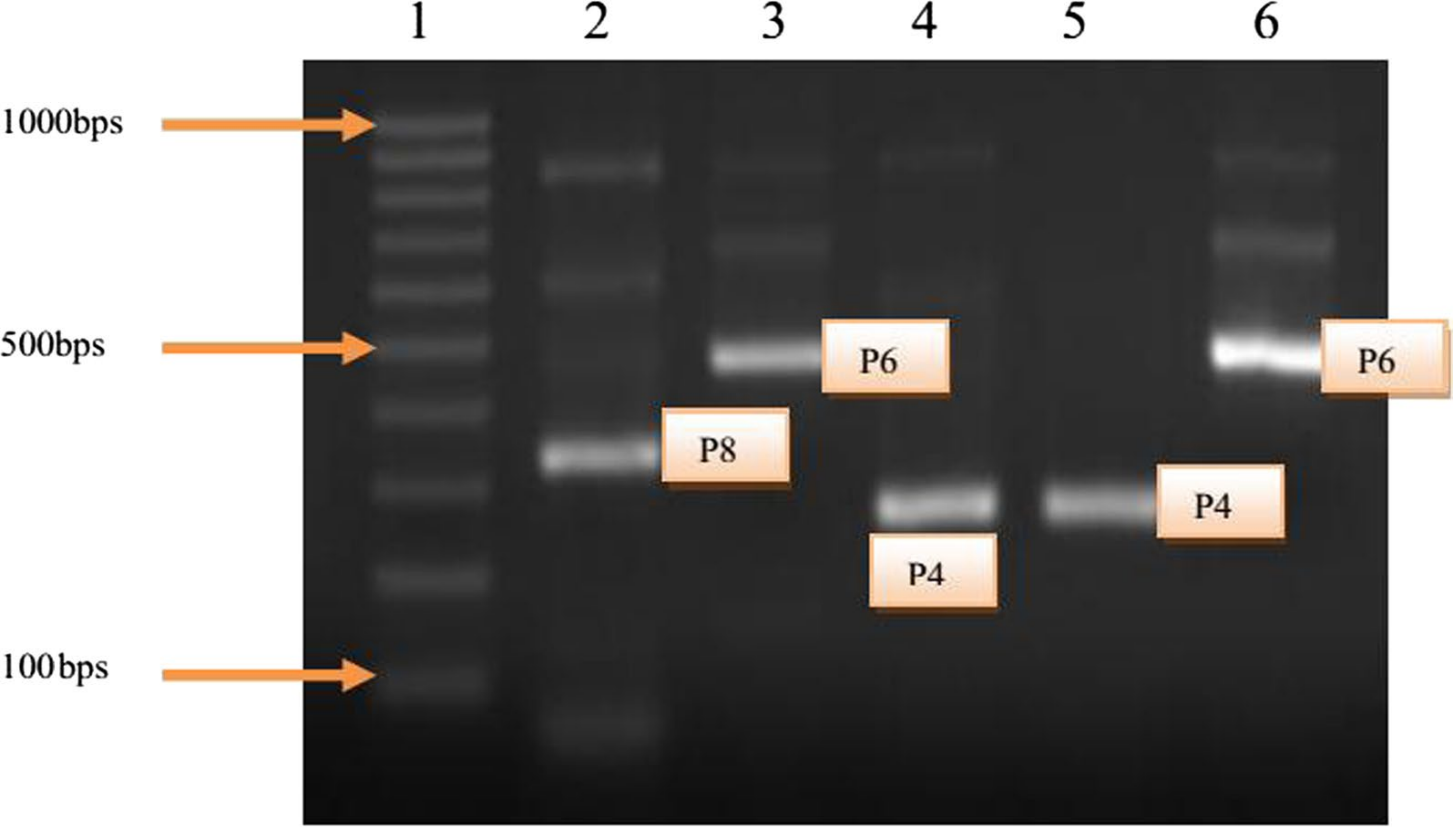
VP4 genotypes as seen under UV light in agarose gel. *Lane 1* 1000 bp marker, *lane 2* P[8] 345 bp, *lane 3* P[4] 483 bp, *lane 4* and *5* p[6] 267 bps, and *lane 6* P[4] 483 bps

Only three P types were shown to be prevalent in Mukuru slums: P[4]—4 (11.4%), P[6]—8 (22.9%), and P[8]—21 (60%), (Fig. 4). P[6]/P[8] mixed types were detected in two samples (5.7%) (Table 2), while no P [9] or P[10]-type viruses were detected during the survey. Two samples (5.4%) were untypeable. The incidence of each type was seen to vary from month to month, with P[6] occurring most frequently in January, while an increase in P[8] was noticed in August and February. The incidence of P[4] was high in January (Table 2).

### Combination of G and P types

**Fig. 5:**
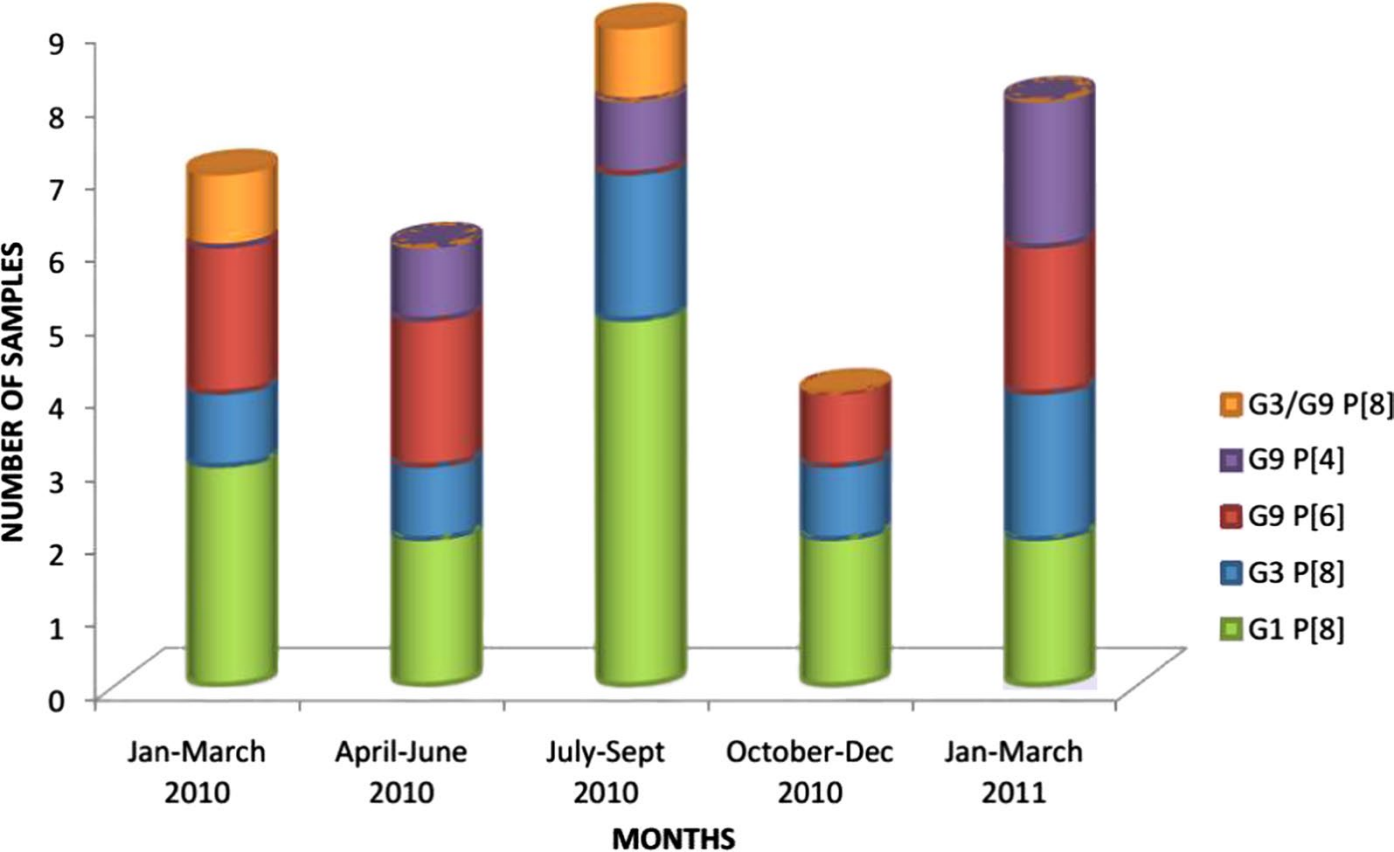
Graphic illustration showing the incidence and diversity of rotavirus genotypes for a period of 15 months in Mukuru slums Nairobi county. G1 P[8] had the highest prevalence (41.2%). The other types, namely, G3 P[8], G9 P[4], G9 P[6], and mixed types (G3/G9 P[8]) were less frequent (20.3, 11.8, 20.3 and 5.9%, respectively)

During the typing assays it was observed that a specific G type could always coexist with a certain P type; namely, G1 and G3 always coexisted with P[8], while G9 was found to associate with P[6] or P[4]. The incidence of each genotype varied considerably for the 15 months (Fig. 5). Overall, G1 P[8] was recorded as the most common (41.2% of all doubly typed viruses). The other types, namely, G3 P[8], G9 P[4], G9 P[6], and mixed types (G3/G9 P[8]) were less frequent (20.3, 11.8, 20.3 and 5.9%, respectively). It was noted that G1 P[8] was the most predominant type collected from both the male and female children.

A marked increase in the number of rotavirus positive samples was observed in July and August. There was an increase in genotype G1P[8] while a decrease was noted in genotype G9 P[6] as confirmed by data shown in Fig. 5. During the cold season, 35.7% of G1 P[8] types were collected in this study.

## Discussion

The results of this study describe the first genotyping results for rotavirus strains associated with acute diarrhoea in infants and young children in Mukuru slums Kenya. The significance of determining genotypes of circulating rotaviruses has become increasingly recognized [11] and RT-PCR has been shown to be the most sensitive assay for determining genotypes [12]. Two major RNA patterns designated as short and long profiles were detected by PAGE, the long strain being dominant (32/40; 80%). The short strains were detected in eight samples (20%). This corresponds to a study carried out in Maua Meru north, Kenya on children below 5 years of age which showed that the long electropherotypes dominated at 80% [13]. This study showed that the long strains existed throughout the year whereas the short strains were found in existence only between April and December. These results confirm the findings of other studies carried out in South Africa and Hungary [14].

The monthly distribution of the long electropherotypes appeared to be random and no apparent seasonal variations could be detected. The short profiles were more prevalent during cold and wet months and were not observed in dry months of the study period. This data shows similar epidemiologic pattern to what have been found in the African developing countries [15]. The distribution of long RNA electropherotypes did not appear to vary with age and it occurred in all age ranges (1 month to 5 years old), while the short RNA profiles were only identified among children aged up to 2 years old (Table 1).

The majority of the genotypes identified in this study fall within the range of globally common strains. As expected from similar surveys carried out worldwide, G1 was the most predominant type detected in each of the 13 months in Mukuru; identified in 40.5% of the G types. Also, study carried out previously in Kenya [13] had indicated G1 as the most predominant. Other studies carried out in India from 1996 to 2001 [16] and in South Korea during 2002–2003 [17] showed that G1 was the most common prevailing genotype. However, in another study done in 2009 [18] on rotavirus infections among HIV-infected children in Nairobi Kenya, G3 was the predominant genotype and emerged as a significant type with a detection rate of 40%. This seems to indicate a notable relative shift in the prevalence of circulating viruses, which should be monitored over the coming years.

After the P-typing assays, it was discovered that the Mukuru results matched those observed in studies from seven other countries in that the P[8] type prevailed. It was also observed that its relative incidence over the 15 months varied, with a detection peak in the month of August. Subsequently, P[6] was the second in detection after P[8] and appeared in 22.9%, followed by P[4] which was detected in 11.4% of the P types. The slightly lower efficiency relative to P typing can be partially attributed to the RNA degradation from some of the earlier isolates, since the G-typing assays were completed first.

Only five samples (6.7%) could not be assigned a G or a P type, which suggests that the incidence of unusual genotypes among Mukuru isolates is rare. This figure is low when compared to the data from other studies in which, approximately 30% of rotavirus positive stool specimens could not be P or G typed [9]. The reason for the occurrence of untypeable strains is unclear. It is possible that inhibitors present in extracted RNA specimens prevent enzyme function in RT and PCR steps of the VP7/VP4 typing assays, hence no amplification. In a similar fashion, subtle changes in the primer binding regions could prevent typing.

The use of more recently developed VP4/VP7 first round primers may improve P typing rates [19]. The third possibility is that these strains may present truly novel G and P types, and only sequence analysis of their PCR products would confirm this. Previous studies have shown that characterization of non-typeable strains have occasionally led to the identification of novel G and P genotypes [7]. The untypeable strains in this study may be due to the emergence of new genotypes and thus further research by characterization and sequence analysis should be considered in the near future.

Regarding G–P combinations, the major ones identified during the study were G1 P[8] and G3 P[8] which are both globally common combinations. In addition, G9 P[6] was also identified at a higher rate but it is not commonly encountered worldwide [9]. This would seem to indicate a significant genotypic shift, which will be of major importance for future studies carried out in Mukuru slums. The VP4 P[6] protein was traditionally thought to be associated with asymptomatic neonatal disease [20]. However, in this study it was identified in young children with acute disease, similar to that reported in other recent studies conducted in Africa and Asia [21]. The accessibility of molecular typing methodology has enhanced the knowledge about G and P genotype diversity and in this study, the rarely seen G9 P[4] and G3/G9P[8] combinations were also identified, further highlighting the ability of rotaviruses to undergo re-assortment [7]. The detection of rotaviral genotypes for the first time in children below 5 years from Mukuru slums, and the indication that the prevalence of certain genotypes may change over a rotavirus season is significant and mirrors observations from studies in other developing countries [22].

## Conclusions

The study concluded that both the short and long strains of rotavirus were in circulation among children in Mukuru slums, with the long strain leading in both the rate of infection and age distribution with serotypes G1, G3 and G9 in combination with P[4], P[6] and P[8] being the main genotypes circulating in children below 5 years in Mukuru slums. The untypeable strains in this study may be due to the emergence of new genotypes hence, further research by characterization and sequence analysis should be considered in the near future to determine whether they are novel isolates or otherwise. Introduction of universal rotavirus vaccines would change the distribution of rotavirus genotypes associated with severe rotavirus-associated acute gastroenteritis.

### Limitations

1. Lack of funds—the study had not won any grant and therefore financing it was a major limitation.

## Abbreviations

cDNA: complementary deoxyribonucleic acid
DNA: deoxyribonucleic acid
EIA: enzyme immunoassay
ELISA: enzyme-linked immunosorbent assay
GIT: gastrointestinal tract
PAGE: polyacrylamide gel electrophoresis
PCR: polymerase chain reaction
RNA: ribonucleic acid
RT-PCR: reverse transcription polymerase chain reaction
RV: rotavirus
RVGE: rotavirus gastroenteritis
UV: ultraviolet
VP4: viral protein 4
VP7: viral protein 7
WHO: World Health Organization
